# Attachment to Pets Moderates Transitions in Latent Patterns of Mental Health Following the Onset of the COVID-19 Pandemic: Results of a Survey of U.S. Adults

**DOI:** 10.3390/ani11030895

**Published:** 2021-03-21

**Authors:** Shelby E. McDonald, Kelly E. O’Connor, Angela Matijczak, Camie A. Tomlinson, Jennifer W. Applebaum, Jennifer L. Murphy, Barbara A. Zsembik

**Affiliations:** 1 School of Social Work, Virginia Commonwealth University, 1000 Floyd Ave, Richmond, VA 23284, USA; matijczaka@vcu.edu (A.M.); tomlinsonc2@vcu.edu (C.A.T.); murphyjl4@vcu.edu (J.L.M.); 2 Department of Psychology, Virginia Commonwealth University, 806 West Franklin St., Richmond, VA 23284, USA; oconnorke2@vcu.edu; 3 Department of Sociology and Criminology & Law, University of Florida, 3219 Turlington Hall, Gainesville, FL 32611, USA; jennyapplebaum@ufl.edu (J.W.A.); zsembik@ufl.edu (B.A.Z.)

**Keywords:** pets, dogs, cats, mental health, COVID-19, latent transition analysis

## Abstract

**Simple Summary:**

The COVID-19 pandemic has contributed to elevated rates of mental health problems and distress among the U.S. population. Pets may be an important source of social support to combat social isolation. This cross-sectional study used latent profile analysis to identify subgroups of U.S. pet owners based on their perceived mental health symptoms prior to and after the onset of the pandemic. Latent transition analysis was used to determine the stability of subgroup membership and examine the effect of attachment to pets on transition probabilities. Five subgroups were identified: low symptoms, mild symptoms, moderate symptoms, high symptoms, and severe symptoms. Evidence of moderation was found, *X*^2^(16) = 41.47, *p* = 0.04. Specifically, results indicated that attachment to pets functioned as a protective factor for individuals exhibiting moderate and high levels of mental health symptoms, as above average attachment to pets was associated with greater odds of transitioning to a less severe symptom profile. However, individuals with severe symptom profiles and high attachment to pets fared worst in the context of COVID-19 restrictions. This study has important implications for future research investigating the role of pets on mental health and for those providing services to pet owners during the COVID-19 pandemic.

**Abstract:**

This cross-sectional study examined whether, and to what extent, attachment to pets was associated with changes in latent patterns of adults’ perceived mental health symptoms during the COVID-19 pandemic (*n* = 1942). We used latent transition analysis to determine the stability of subgroup membership pre- and post-COVID and the effect of attachment to pets on transition probabilities. Mental health before COVID-19 was measured retrospectively. Five subgroups were identified: low symptoms, mild symptoms, moderate symptoms, high symptoms, and severe symptoms. Among individuals in the moderate and high symptoms subgroups, those who reported high attachment to pets generally had greater odds of transitioning to a less severe symptom profile (*OR* = 2.12) over time than those with low attachment to pets (*OR* = 1.39). However, those who had a severe symptom profile and high attachment to pets had lower odds of transitioning to a less severe symptom profile (*OR* = 0.30) and higher odds of maintaining a severe symptom profile (*OR* = 3.33) than those with low attachment to pets. These findings suggest that the protective and risk effects of attachment to pets differ based on individuals’ psychological symptom patterns across multiple indicators. We discuss the implications of these findings for research, policy, and practice.

## 1. Introduction

As of February 2021, coronavirus disease (COVID-19) has infected approximately 28 million people in the U.S. and contributed to more than 500,000 deaths, a rate higher than any other country in the world [1,2]. Research examining the impact of the pandemic on psychological health is rapidly developing. Studies to date suggest that the experience of living through this unprecedented public health crisis is a new type of complex trauma characterized by fear and the threat of future infection and/or death; further, this stress is exacerbated by associated economic stressors, disturbance in routines, isolation, and related secondary traumas [3]. Unsurprisingly, there is increasing evidence that the pandemic has contributed to elevated rates of mental health problems and distress among the U.S. population, including increased levels of anxiety and depression symptoms, substance use, and suicidal ideation among adults [4,5,6,7,8]. For example, Holman et al. [6] found that as rates of COVID-19-positive cases and deaths increased across the U.S, there was a corresponding increase in COVID-19-related acute stress and depressive symptoms among U.S. adults. These findings are consistent with studies on associations between COVID-19-related stress and mental health worldwide [6,9,10].

### 1.1. Animal Companions and the COVID-19 Pandemic

Although the role of companion animals in helping people cope with social isolation and stress has received tremendous attention in mainstream media since the onset of COVID-19-related social distancing guidelines [11,12,13], only a small proportion of studies on mental health during the pandemic have considered pet ownership and/or related aspects of human–animal interaction (HAI) as a risk *and* protective factor for psychological adjustment during this public health crisis. More than half of adults in the U.S. live with at least one pet [14], and the rate of pet ownership is expected to increase due to the popularity of pet adoption, buying, and fostering since the emergence of COVID-19 [15,16]. Research to date documents several social, psychological, and health benefits of living with pets during the pandemic; for example, evidence from recent studies suggests that pets improve quality of life [17], enhance mood [18], encourage physical activity [18], and provide comfort and stress relief via touch [19]. Additionally, a study investigating changes in mental health and loneliness prior to and after the COVID lockdown in the UK found that pet owners reported less deterioration in mental health and smaller increases in loneliness [20].

At the same time, studies also document stressors associated with caring for pets amidst the pandemic. A study based in the U.S. found that pet owners reported several forms of caretaking stress, such as the inability to socialize their pet, difficulties obtaining resources (e.g., food, litter, medication), and managing behavioral issues (e.g., barking, attention-seeking) that disrupted telework routines [21]. Further, a study of pet owners living in the U.S. found that owners who were more attached to their pet were more likely to report delaying COVID testing or treatment due to worries about their pet’s welfare [22]. Additionally, some pet owners reported concerns that their pet might contract COVID-19 and pass the virus to human members of the household [18,23].

These studies highlight the important role that pets may play in coping with and/or exacerbating psychological stress during the COVID-19 pandemic. However, there is a notable scarcity of empirical studies investigating how relationships with, or the presence of, a pet impacts changes in mental health. In particular, no study to date has examined the role of attachment to pets in relation to changes in *patterns* of mental health symptomatology prior to and after the onset of the pandemic using multiple indicators of adjustment (e.g., depression, anxiety). Prior studies of mental health and attachment to pets suggest that higher levels of emotional dependence on pets, particularly among dog and/or cat owners, is associated with increased levels of mental health symptomatology (e.g., depression, anxiety) [24,25]; such evidence must be considered alongside other evidence that pets (i.e., especially dogs and cats) may reduce stress responses via physical contact or petting [19,26,27]. Thus, there is a significant need to understand how individuals’ attachment to pets influences changes in psychological adjustment in the context of the ongoing COVID-19 public health crisis.

### 1.2. Person-Centered Approaches to Examining Relations between Mental Health and HAI

Studies consistently demonstrate that comorbidity of mental health disorders is related to higher symptom severity and poorer overall quality of life [28,29,30,31,32]. Person-centered approaches, such as latent profile analysis (LPA), provide a framework to consider the multidimensionality of mental health symptomatology and identify subgroups of people characterized by similar symptom profiles, such as comorbidity of anxiety and depression symptoms [33,34]. LPA allows for the examination of patterns of psychological symptoms based on how individuals respond to a specific set of indicators (e.g., subscales of a mental health measure). Latent transition analysis (LTA) extends LPA by estimating the probability that subgroup membership (i.e., the probability that patterns in the construct, such as mental health symptomatology) changes over time [35]. These approaches also allow for the identification of factors that influence change in subgroup membership over time (e.g., attachment to pets and demographic factors), which can provide additional information regarding the susceptibility for risk and resilience over time and provide targets for interventions [36,37]. 

Despite the utility of person-centered methods for informing targeted interventions, few studies have applied person-centered approaches to the study of psychological adjustment among adults; this paucity applies to studies prior to and/or during the COVID-19 pandemic. Generally, studies using person-centered approaches to examine patterns of psychological adjustment prior to the pandemic have identified multiple subgroups defined by profiles of comorbidity (e.g., multiple externalizing disorders) [38]. As reviewed in a recent study by Kim and Eaton [38], some researchers have argued that four classes are sufficient to represent patterns of common mental disorders in the U.S. population [39,40], whereas others have argued for the utility of five [41], seven [42,43], or nine subgroups [44]. However, due to methodological inconsistencies across studies (e.g., number of indicators used in the analysis, fit indices used to determine the optimal number of subgroups, measures of mental health [e.g., lifetime vs. 12-month diagnosis]), it is difficult to compare and synthesize results across studies [38]. Moreover, few studies have used continuous (i.e., in LPA) rather than categorical (often binary) indicators. The use of continuous indicators in LPA provides the model with more information about each individual and thus can allow for greater precision in identifying homogeneous subgroups of individuals within a population. 

We are aware of only one study that has examined profiles of psychological adjustment during the pandemic using multiple continuous indicators of mental health symptoms. In a large study of Argentinian adults, Fernandez et al. [45] examined patterns of psychological adjustment using nine indicators on the Brief Symptom Inventory (BSI) [46]: somatization, anxiety, phobic anxiety, obsessive-compulsive, interpersonal sensitivity, depression, hostility, paranoid ideation, and psychoticism. They found that participants were best characterized by one of three latent profiles of psychological adjustment: low, mild, and severe levels of symptoms; most individuals were classified in the mild (40.9%) and severe (41.0%) classes [45]. Notably, the mild and severe classes reported elevated levels of phobic anxiety, obsessive-compulsive, and hostility symptoms, in addition to anxiety, depression, and general distress. This finding is important because many studies of COVID-19-related mental health have neglected to examine dimensions of psychological stress beyond depression and anxiety. Moreover, some studies indicate that U.S. individuals with preexisting mental health conditions are at greatest risk for increases in psychological distress during the pandemic [6]. Considering evidence that most individuals experience symptoms of more than one psychological disorder at a single time point, the application of person-centered methods to the study of COVID-19-related psychological adjustment and related risk and protective factors may be particularly useful [38,41]. 

### 1.3. Current Study

The application of person-centered approaches (i.e., LPA, LTA) to the study of HAI in the context of COVID-19 has the potential to yield more targeted information regarding how and for whom HAI may provide the most benefits or risks [36]. The current cross-sectional study examines whether attachment to pets influences changes in individuals’ pattern of perceived psychological adjustment before and after the onset of the COVID-19 pandemic. Notably, self-reported psychological adjustment was measured during the pandemic such that participants were asked to rate their current (during pandemic) and past (pre-pandemic) mental health symptoms. Identifying underlying subgroups of individuals based on their patterns of psychological adjustment and how these patterns changed over time in relation to HAI provides a more holistic picture of psychological adjustment in comparison to variable-centered methods which rely on group means and covariance, as well as the assumption that the sample represents a uniform population [47]. Although it is hypothesized that humans living in households with companion animals have experienced mental health benefits from the companionship of their pets during the COVID-19 pandemic, no study to date has examined whether, and to what extent, attachment to pets is associated with changes in adults’ patterns of mental health symptoms during the early stages of this public health crisis. To address this gap in the literature, the goals of the current study are to: (a) identify latent profiles (i.e., subgroups) of U.S. pet owners’ self-perceived psychological adjustment (i.e., BSI subscale scores) prior to and during the early stages of the pandemic, (b) examine the stability of subgroup membership (i.e., latent transitions; the probability of transitioning to a subgroup with a more or less severe symptom profile versus staying in the same subgroup over time), and (c) examine the extent to which attachment to pets (i.e., cats, dogs) predicts transitions in subgroup membership prior to and during the early stages of the pandemic. 

Due to the scarcity of studies that have utilized person-centered approaches to investigate changes in psychological adjustment during the COVID-19 pandemic, these analyses were largely exploratory. However, we were able to make some specific hypotheses based on prior work. Given the findings of Fernandez et al. [45], we expected that we would identify at least three subgroups of psychological adjustment during the pandemic, including a subgroup with a low or “low symptomatology” profile (i.e., little to no symptoms of mental health problems), one with a mild symptom profile, and a subgroup with a severe symptom profile (i.e., symptoms highly elevated across BSI subscales). We also hypothesized that some of those with a less severe symptom profile (e.g., low, mild) at pre-COVID would transition to a more severe symptom profile, which is based on evidence that the pandemic has led to higher rates of depression and anxiety [4,5,6,7,8]. Finally, we expected that attachment to pets would predict changes in psychological adjustment based on prior research documenting both the negative and positive effects of HAI (e.g., [18,21,22]). We were not able to make any more specific predictions about the effects of attachment to pets on transitions in subgroup membership over time given that no prior HAI studies have utilized a person-centered approach to examine changes in psychological adjustment before and after the onset of the COVID-19 pandemic. 

Because prior evidence suggests that people report higher levels of attachment to cats and dogs compared to other animal companion species [48,49,50], we restricted our study to individuals who reported on attachment to pet dogs and cats. In addition, given that sociodemographic factors, such as being younger in age; identifying as a racial/ethnic, gender, and/or sexual minority; having a lower income; and experiencing changes in employment status and wage loss, have been linked to psychological risk in the context of pandemic-related stress, we adjusted for the effects of these factors in our analyses [6,7,8,51]. 

## 2. Materials and Methods

We distributed an anonymous survey on the Internet in the spring and summer of 2020 to understand various aspects of interactions with pets during COVID-19. The Qualtrics software survey link was distributed via social media and other online channels, including Facebook, Twitter, Instagram, Reddit, and various listservs related to companion animals. We utilized convenience sampling and social media recruitment techniques to collect data rapidly, with the goal of disseminating information regarding the impact of COVID-19 as quickly as possible. Respondents were eligible to participate in the survey if they were age 18 or over, currently residing in the U.S., and had at least one pet/companion animal. The survey was available in English and took approximately 30 min to complete. Respondents were not compensated for their participation. Topics included in the survey pertained to interactions with pets during the COVID-19 pandemic, health, and well-being, as well as various measures of social and demographic information. We obtained informed consent from all participants, and this study was approved by the University of Florida Internal Review Board, protocol # IRB202000819. Screening questions and informed consent were mandatory response items; all subsequent closed-ended and open-ended questions were optional and could be skipped.

### 2.1. Participants

We collected 3006 total responses between 6 April and 21 July 2020. For the present study, we restricted our sample to dog and/or cat pet owners who completed a pet attachment measure about either their dog or cat (*n* = 2463). A total of 441 participants were excluded from LPAs due to missing data on all indicators (i.e., BSI subscales). Further, we excluded any respondent from the analysis who had missing data on any covariate or the pet attachment measure, resulting in a final sample of 1942 respondents. Demographic characteristics for our sample can be found in Table 1. Approximately 12.5% of our sample identified as a racial/ethnic minority (e.g., Black/African American, Latino/Latina/Latinx), 3.1% of the sample identified as a gender minority (e.g., transgender, gender non-conforming), and 21.3% of the sample identified as a sexual minority (e.g., bisexual, gay, lesbian). Participant ages ranged from 18 years to 85 years, with an average age of 39.68 years (*SD* = 13.61). More than half of our sample reported owning at least one dog (74.3%); cats were the second most common pet, with 53% of the sample reporting living with at least one cat. Further, in responding to the pet attachment questionnaire, a majority of participants (65.7%) answered questions about a dog they owned, with the remaining 34.3% reporting on a cat. 

### 2.2. Measures

#### 2.2.1. Indicators of Mental Health

Psychological adjustment was measured using the Brief Symptoms Inventory (BSI) [52], a 53-item validated scale to assess mental health. Our study used eight subscales of the BSI: anxiety (6 items; e.g., “nervousness or shakiness inside”), depression (5 items; e.g., “feeling no interest in things”), hostility (5 items; e.g., “having urges to break or smash things”), interpersonal sensitivity (4 items; e.g., “feeling that people are unfriendly or dislike you”), obsessive-compulsive (6 items; e.g., “difficulty making decisions”), phobic anxiety (5 items; e.g., “feeling nervous when you are left alone”), somatization (7 items; e.g., “faintness or dizziness”), and additional items (4 items; e.g., “poor appetite”). We eliminated one item on the depression subscale (i.e., “thoughts of ending your life”). This item was removed at the request of the IRB, as appropriate crisis management would not be feasible due to the online and anonymous nature of the survey. Respondents were asked to indicate the extent to which each item distressed or bothered them both prior to the onset of, as well as during, the COVID-19 pandemic, on a five-point Likert scale from 0 (not at all) to 4 (extremely). Thus, mental health prior to the onset of COVID-19 was measured retrospectively. Scores of each subscale were computed as the mean of the endorsed items. Internal consistency of all subscales was excellent at both time points (α > 0.95).

#### 2.2.2. Attachment to Dogs and Cats

Participants’ level of attachment to pets was assessed using the Lexington Attachment to Pets Scale (LAPS) [53], a 23-item measure of emotional attachment to pet(s). Cat and dog owners indicated their current (during the pandemic) level of agreement on a five-point Likert scale from 1 (strongly agree) to 4 (strongly disagree) with a fifth option (don’t know) to statements such as, “My pet knows when I’m feeling bad,” and “My pet makes me feel happy.” Potential scores on this summated scale ranged from 23 (low attachment) to 92 (high attachment). Internal consistency of the scale in our sample was excellent (α = 0.997). 

#### 2.2.3. Covariates

Sociodemographic information was collected for respondents, including age (in years), race/ethnicity, gender identity, sexual orientation, relationship status, and whether their employment status had changed due to COVID-19. Participants were able to select multiple options for race/ethnicity (i.e., Arab/Arab American, Asian/Asian American, Black/African American, First Nations/Indigenous, Latina/Latino/Latinx, Multiracial/Mixed Race, South Asian/Pacific Islander, White, White Ethnic [Jewish, Italian, Irish, etc.]), gender identity (i.e., male, female, transgender male/transgender man, transgender female/transgender woman, and genderqueer/gender non-conforming), and sexual orientation (i.e., gay, lesbian, bisexual, two-spirit, queer, straight/heterosexual, pansexual, asexual, demisexual, and not sure or questioning). Participants were also given the option to self-describe their race/ethnicity, gender identity, and sexual orientation, and were able to select “prefer not to say” for gender identity. These variables were recoded for analyses: race/ethnicity (1 = White and/or White Ethnic and non-Latinx, 0 = minority race/ethnicity or multiple races/ethnicities) and sexual and/or gender minority (1 = gender minority and/or sexual minority, 0 = cisgender and heterosexual). Participants also indicated their current relationship status (i.e., married or permanently partnered/cohabiting; single, never married nor permanently partnered/cohabiting; divorced; separated; widowed; or prefer to self-describe), which was coded such that: 1 = in a relationship (i.e., married or permanently partnered/cohabiting) and 0 = not in a relationship. Additionally, responses to whether participants’ employment status had changed due to COVID-19 were coded to reflect no change (=0) and change in employment (=1; working from home, laid off or fired, begun a new job, other). Across all variables, responses to the “prefer to self-describe” option were reviewed and, when appropriate, re-coded into an existing category. Otherwise, “prefer to self-describe” and “prefer not to say” responses were coded as missing.

### 2.3. Analytic Plan

**Figure 1:**
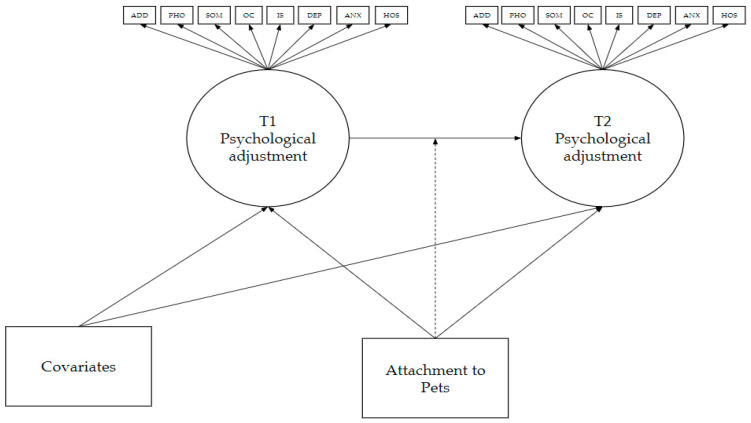
Analytic Model.

All analyses were conducted using Mplus Version 8.5 (Los Angeles, CA, USA) Separate LPAs were conducted to identify subgroups of individuals based on their composite scores on the BSI subscales prior to (T1) and after the onset (T2) of the COVID-19 pandemic (Figure 1). As noted previously, all participants with missing data on the latent profile indicators (i.e., BSI subscales) were excluded from analyses (*n* = 441). Full information maximum likelihood (FIML) was used to address any further missing data. We followed best practices outlined by Masyn [54] for estimating LPA models. For each LPA, we tested four different variance–covariance structures: (a) diagonal class-invariant (i.e., covariance of indicators fixed to zero, variance of each indicator constrained to be equal across subgroups), (b) diagonal class-varying (i.e., covariance of indicators fixed to zero, variance of each indicator is free to vary across subgroups), (c) non-diagonal class invariant (i.e., constrains the indicator variances and covariances to be equal across subgroups), and d) non-diagonal class varying (i.e., variances and covariances allowed to vary across subgroups). The optimum number of subgroups was determined based on theory, group size considerations, and comparison of fit indices. Fit indices included log likelihood, the Akaike information criterion (AIC), Bayesian information criterion (BIC), sample size-adjusted BIC (aBIC), the Lo–Mendell–Rubin likelihood ratio test (LMR-LRT), the Vuong–Lo–Mendell–Rubin likelihood ratio test (VLMR-LRT), and the bootstrap likelihood ratio test (BLRT). Smaller AIC, BIC, and aBIC values indicated better fit, and a significant LMR-LRT, VLMR-LRT, or BLRT indicated an improvement in fit from a model with one fewer subgroup.

Once the best-fitting LPA model was identified at each time point, we examined whether several sociodemographic variables were related to subgroup membership. Specifically, we considered the following sociodemographic variables: age, change in employment status due to the COVID-19 pandemic, race/ethnicity, sexual and gender minority status, and relationship status. We used the three-step manual BCH approach to regress all sociodemographic variables on the latent categorical variable for each time point separately. The overall effect of each variable on latent profile membership was evaluated with a Wald test and the subgroup-specific effects were assessed with odds ratios (ORs) which were calculated within the model constraint command.

Next, we used latent transition analysis (LTA) to address the second and third goals of the current study, namely to determine the stability of subgroup membership over time and the effect of attachment to pets on transition probabilities. All covariates that were significant predictors of latent profile membership at any time point in the previous step were regressed on to both latent categorical variables in the LTA model. Values for age and attachment to pets were divided by 10 in analyses to minimize estimation issues resulting from large variances. As with the LPAs, all other missing data were handled using FIML. To determine whether there was a significant effect of attachment to pets on transitions in subgroup membership, we conducted a likelihood-ratio chi-square difference test comparing a model (H0) in which the predictor was regressed on both latent class variables with a model (H1) in which the predictor moderated the relation between the latent class variables. We examined logistic regression coefficients to determine the specific effects of attachment to pets on transitions between subgroups over time. 

We also calculated the odds of moving to a subgroup with a *more severe* symptom profile (versus staying in the same subgroup over time) and moving to a subgroup with a *less severe* symptom profile (versus staying in the same subgroup over time) between those with below average attachment to pets and those with above average attachment to pets. This was done through several steps. First, we used the LTA calculator in Mplus to determine the probability that each subgroup at T1 would transition to each subgroup at T2 for those with above average attachment to pets, then repeated this process to determine the transition probabilities for those with below average attachment to pets. We then calculated the odds of moving to a more severe subgroup and the odds of moving to a less severe subgroup (versus staying in the same subgroup) for those with above average attachment to pets, then repeated this process to calculate the same odds among those with a below average attachment to pets. Finally, we divided the odds of moving versus staying for those with below average or above average attachment to pets. We were not able to calculate these ORs within the model constraint function of Mplus (e.g., some of the transition probabilities were zero, which led to estimation issues in the Mplus framework). Thus, because the ORs were calculated manually, we were not able to determine the significance level of the ORs.

## 3. Results

### 3.1. Descriptive Statistics

We ran independent samples t-tests and chi-square tests to investigate differences between respondents included in the analysis and respondents excluded due to missing data. There were two significant differences: interpersonal sensitivity post-COVID [*t* (1996) = 2.02, *p* = 0.04] and attachment to pets [*t* (733.96) = −3.72, *p* < 0.001]. However, the effect size of both *t*-tests was small (interpersonal sensitivity: Cohen’s *d* = 0.237, 95% CI: [0.01, 0.47]; attachment to pets: Cohen’s *d* = −0.19, CI: [−0.30, −0.08]). This suggests that the degree to which mean scores differed between those included and excluded is not a meaningful difference [55]. As reported in Table 2, pre-COVID BSI subscales exhibited moderate to high correlations with one another (*r*s range from 0.35 to 0.68) as well as low to high correlations with post-COVID BSI subscales (*r*s range from 0.20 to 0.73). Attachment to pets was significantly correlated with BSI subscales, but the associations were weak for both pre-COVID (*r*s range from 0.07 to 0.12) and post-COVID (*r*s range from 0.06 to 0.13). Employment change was significantly and weakly associated with all pre-COVID subscales except the somatic and additional scales (*r*s range from 0.06 to 0.13) and all post-COVID subscales (*r*s range from 0.05 to 0.15). Race/ethnicity was significantly and weakly associated with post-COVID hostility and pre-COVID obsessive-compulsive, depression, and hostility symptoms (*r*s range from −0.05 to −0.8). Sexual and/or gender minority identity exhibited significantly low correlations with all pre-COVID subscales (*r*s range from 0.12 to 0.21), post-COVID subscales (*r*s range from 0.10 to 0.22), and change in employment (*r* = 0.10). Being in a relationship exhibited significant, but weak correlations with age (*r* = 0.10); sexual/gender minority identity (*r* = −0.14); attachment to pets (*r* = −0.06); pre-COVID obsessive-compulsive, interpersonal sensitivity, depression, anxiety and additional items (*r*s range from −0.06 to −0.19); and all post-COVID subscales except hostility and phobic anxiety (*r*s range from −0.06 to −0.18). Table 2 displays means, correlations, and proportions for all continuous study variables. Notably, sample means for BSI subscales increased slightly from pre- to post-COVID.

### 3.2. Latent Profile Analyses

Examination of the fit statistics across models specifying between one and seven latent classes provided the clearest support for a five-class model with a diagonal (i.e., covariances among indicators fixed to 0) invariant (i.e., variances of each BSI subscale constrained across classes) structure for both pre-COVID psychological adjustment (see Table 3) and post-COVID psychological adjustment (see Table 4). Means were free to vary in each variance–covariance structure that was tested. For both pre- and post-COVID diagonal-invariant models, the BIC and adjusted BIC continued to decrease across *k*-class solutions. Examination of a scree plot for models at each time point revealed that the magnitude of the decrease leveled off after the four- or five-class diagonal-invariant model. Further, the significance of both the LMR-LRT and VLMR-LRT at each time point indicated that the five-class model significantly improved upon the fit of the four-class diagonal-invariant model. The non-diagonal class-invariant four-class models were considered as candidate models because they had lower AIC, BIC, and aBIC values than the five-class diagonal-invariant models. However, the pre- and post-COVID five-class diagonal-invariant models exhibited greater separation compared with the non-diagonal-invariant four-class model at each time point, as indicated by significant differences across subgroups on each indicator (*p*s < 0.001). The entropy and condition numbers of each five-class diagonal class-invariant model were also adequate.

**Figure 2:**
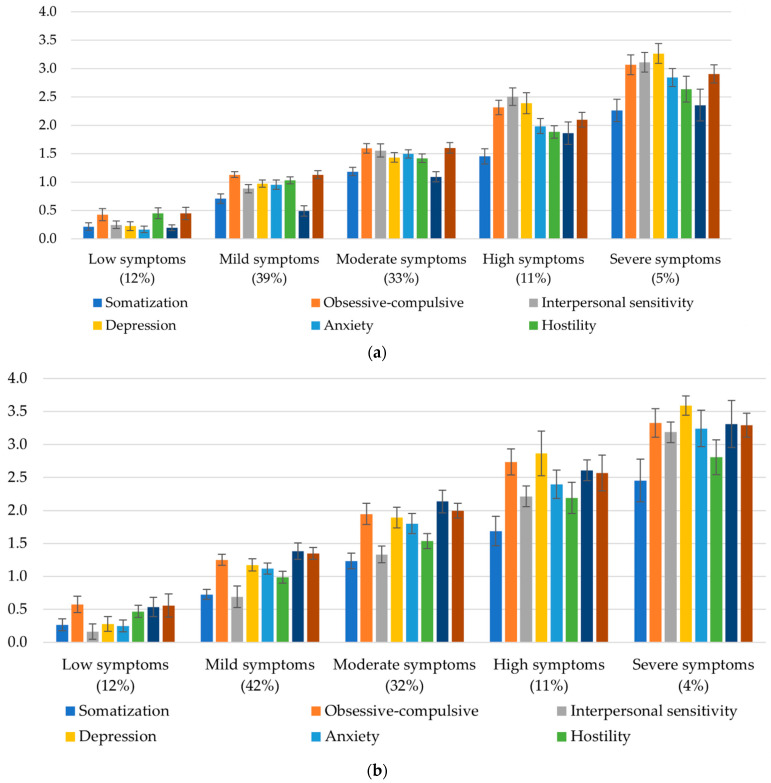
Subgroup means for pre- and post-COVID BSI subscales with 95% confidence intervals. *Y*-axis values represent mean scores for each subscale. (**a**) Subgroup means for pre-COVID BSI subscales; (**b**) subgroup means for post-COVID BSI subscales.

For the pre-COVID five-class diagonal-invariant model, examination of BSI subscale means within each class revealed several patterns (Figure 2a). The “low symptoms” subgroup (12%) had mean levels of each indicator between 0.16 and 0.45, suggesting that individuals in this subgroup generally exhibit good psychological adjustment. The “mild symptoms” subgroup made up the largest proportion of the sample (39%) and exhibited higher mean levels of each indicator than the low symptoms subgroup (*p*s < 0.001), with means ranging from 0.49 to 1.31. The mild symptoms subgroup also exhibited significantly lower mean levels of each indicator (*p*s < 0.001) than the other three subgroups: “moderate symptoms” (33%; *M*s ranging from 1.09 to 1.60), “high symptoms” (11%; *M*s ranging from 1.46 to 2.50), and “severe symptoms” (5%; *M*s ranging from 2.26 to 3.26). Overall, indicator means differed significantly across all subgroups without exception (*p*s < 0.001). As shown in Figure 2b, the post-COVID five-class diagonal-invariant model revealed a similar pattern to that of the pre-COVID model, including (a) a “low symptoms” subgroup (12%; *M*s ranging from 0.16 to 0.57), (b) a “mild symptoms” subgroup (42%; *M*s ranging from 0.69 to 1.38), (c) a “moderate symptoms” subgroup (32%; *M*s ranging from 1.23 to 2.14), (d) a “high symptoms” subgroup (11%; *M*s ranging from 1.69 to 2.87), and (d) a “severe symptoms” subgroup (4%; *M*s ranging from 2.46 to 3.59). Similar to the pre-COVID LPA model, indicator means differed significantly across all subgroups (*p*s < 0.001). Further, the subgroup proportions appeared to be relatively consistent over time, with no more than 3% difference between the proportions of each subgroup at pre- and post-COVID. Notably, however, the indicator means in the post-COVID LPA model are somewhat elevated relative to the pre-COVID LPA model, suggesting a broad increase in psychological maladjustment.

### 3.3. Latent Transition Analysis

A latent transition analysis with covariates was conducted to examine the stability of subgroup membership over time. We conducted a chi-square difference test using the log likelihood to determine whether the null model in which the indicator means were constrained across time improved upon the fit of the alternative model in which the means were freely estimated. Results did not find support for measurement invariance across time, *X*^2^(40) = 1766.0, *p* < 0.001. Thus, indicator means were allowed to vary across time.

Approximately 80% of participants remained in the same subgroup between pre-and post-COVID (see Table 5). Transitions to subgroups with poorer psychological adjustment occurred among 11% of participants. For instance, 2% of the low symptoms subgroup, 5% of those with mild symptoms, 4% of those with moderate symptoms, and slightly under 1% of those with high symptoms transitioned to a subgroup with poorer psychological adjustment following the onset of the COVID-19 pandemic.

A latent transition analysis was conducted to examine attachment to pets as a predictor of transition probabilities. Attachment to pets significantly moderated the relation between latent profile membership over time, *X*^2^(16) = 41.47, *p* = 0.042. This was primarily evident for those with moderate, high, or severe psychological adjustment profiles pre-COVID, as indicated in Table 5. Among individuals in the moderate and high symptom subgroups, those who reported high attachment to pets generally had greater odds of transitioning to a less severe symptom profile over time than those with low attachment to pets (*OR*s = 2.14 and 1.39, respectively). In contrast, those who had a severe symptom profile and high attachment to pets had lower odds of transitioning to a less severe symptom profile (*OR* = 0.30) and higher odds of maintaining a severe symptom profile over time (*OR* = 3.33) compared to those with low attachment to pets.

## 4. Discussion

The goal of this study was to utilize a person-centered approach to identify patterns of psychological adjustment before and during the beginning months of the COVID-19 pandemic among a sample of adult pet owners living in the U.S. Further, we aimed to investigate how attachment to pets may have influenced changes in mental health symptom patterns as people adjusted to following public health guidelines. Although this study was largely exploratory, we hypothesized that the pre-COVID LPA would identify at least three subgroups of psychological adjustment, including low, mild, and severe symptom profiles. This hypothesis was supported as our LPA analyses identified five subgroups of mental health symptomatology: low symptoms, mild symptoms, moderate symptoms, high symptoms, and severe symptoms. These subgroups share some overlap with the results of Fernandez et al. [45]. Though Fernandez et al. [45] identified only three subgroups (i.e., low, mild, and severe symptoms), the patterns they identified are similar to the low, mild, and severe symptom profiles identified in the current study. Moreover, the patterns identified in the current study are similar to the findings of Fernandez et al. [45] in that they are defined by severity level of symptomatology, rather than comorbidity of symptoms of specific mental health disorders. Additionally, we identified two subgroups that Fernandez et al. [45] did not, namely the moderate and high symptom profiles. Given that LPA is sample-specific, the differences in the patterns identified may be related to the timing of data collection or cultural factors related to mental health or rates of COVID-19, as Fernandez et al. [45] collected data only during the first half of April 2020 and focused on a sample of Argentinian adults. In addition, Fernandez et al. [45] used a Spanish language version of the BSI; prior studies suggest that the factor structure of the BSI may differ across language versions [56,57,58,59], and that there are cultural differences in the expression of mental health symptoms across regions of Argentina [60].

Second, we hypothesized that some of those with a less severe pre-COVID symptom profile would transition to a more severe post-COVID symptom profile. Although we found that an estimated 11% of participants transitioned to a subgroup with poorer psychological adjustment (e.g., well-adjusted to mild, moderate to severe), the majority of our sample (80%) remained in the same subgroup and did not transition to a more or less severe subgroup. Although pre-COVID psychological adjustment was measured retrospectively in the current study, prior studies have found evidence of an increase in mental health symptoms during approximately the same time period as our data were collected [4,7,8]. In many ways, the results of the current study corroborate these findings, as it is truly significant that an estimated 1 in 5 cat and/or dog owners in our sample experienced a change in their psychological adjustment following the onset of the COVID-19 pandemic, and just over half of these individuals experienced worsening psychological adjustment. Notably, prior studies have used a variable-centered approach to examine psychological adjustment over time and thus have examined changes at the population level rather than the person level (i.e., symptoms increased *overall* among participants). Thus, the findings of our study highlight the importance of utilizing a person-centered approach in understanding the nuances underlying changes in psychological adjustment during the COVID-19 pandemic. Additionally, whereas the current study examined multiple indicators of psychological adjustment, most prior studies have focused solely on depression or anxiety, which are important indicators but do not provide a holistic picture of one’s psychological adjustment. Lastly, pre-COVID psychological adjustment was based on participants’ retrospective self-report in the current study, which may have affected our findings and is discussed further in the limitations section.

We found that attachment to pets moderated the transitions between latent profiles, supporting our third hypothesis. In particular, participants who were highly attached to their pet and initially had moderate or high symptom profiles were more likely than those with low attachment to pets to transition to a less severe symptom profile. This suggests a protective effect of attachment to pets on the change in psychological adjustment during this stressful period. A study conducted by Ratschen et al. [20] during the COVID-19 quarantine in the UK found that pet owners experienced less deterioration in mental health when compared to non-pet owners. Our findings build on this study by suggesting that the attachment individuals feel with their pet is also an important consideration in understanding the mechanisms through which pets may buffer the impact of COVID-19 stressors on mental health decline. 

In contrast, for participants in the severe symptom subgroup, high levels of attachment to pets decreased the likelihood of transitioning to a less severe symptom profile, as compared to those with low levels of attachment to pets. This suggests that, for those with severe psychological maladjustment, strong attachment to a pet may actually cause additional psychological stress during COVID-19 and prevent mental health from improving. It is possible that burdens associated with pet ownership during this time (e.g., obtaining resources, finding alternative caretakers in case of COVID infection) [21] may be an especially impactful form of stress for individuals struggling with severe psychological maladjustment. Additionally, it is important to consider the potential impact of social isolation caused by COVID-19 public health restrictions. Previous studies have found that social support is negatively related with anxiety and depression during the COVID-19 pandemic [61,62]. A study conducted by Matijczak et al. [24] found that deriving comfort from pets exacerbated the relation between exposure to microaggressions and depressive symptoms, but only when participants concurrently reported low levels of social support. It is possible that human forms of social support might be important factors to consider for pet owners with severe psychological maladjustment, as caring for pets may add stress that negatively impacts mental health. It may also be that these individuals were already facing numerous stressors prior to the pandemic and lack resources to cope with these, much less any additional stressors. As a result, these individuals may be at risk for other negative outcomes associated with the pandemic (e.g., difficulty finding and maintaining employment).

### 4.1. Limitations

In addition to the strengths of this study, there are several limitations. The current study utilized convenience sampling and social media recruitment techniques to collect data rapidly, with the goal of disseminating information regarding the impact of COVID-19 as quickly as possible. There is evidence that social media-based recruitment may lead to unrepresentative samples compared to other methods of recruitment [63]. Because a majority of our study sample identified as White non-Latinx, heterosexual, and cisgender women, replication of this study with more representative samples is necessary. Our study also exclusively focused on individuals reporting on dogs or cats as a pet. Thus, the findings of this study may not generalize to those with other pets. The retrospective recall of mental health symptomatology is another limitation, as research suggests this can lead to over- or under-reporting of past experiences [64]. Moreover, considering that each participant may have assessed the time point for “pre-pandemic” mental health differently, and that our data were collected over a period of 3.5 months, variability in the data limits the reliability and validity of our results. Our moderation analysis was also compromised by this aspect of our methodology, as attachment to pets was assessed after the onset of the pandemic, and, due to concerns about participant burden, we were unable to account for changes in attachment or other important factors that may have impacted attachment to pets during this time period (e.g., length of time with the pet, pet temperament and behavior). Another limitation was examining only one form of HAI (i.e., attachment to pets). Investigating other domains of HAI (e.g., comfort from companion animals, pet-related stress) may provide additional insights in future research, particularly when considering ceiling effects in the bond people report to their pets. Our “low attachment” group was derived from the distribution of our sample, and may not necessarily reflect low levels of attachment to pets in the general population. It is also important to consider the limitations of using person-centered approaches such as LPA. Subgroups determined by the analyses may miss other latent, or hidden, groups, or develop subgroups within the sample that do not actually exist or are indistinguishable in the population [65]. Person-centered approaches such as LPA, due to the exploratory nature, are limited in terms of generalizability and whether the groupings from this study are meaningful outside of the current sample [66].

### 4.2. Future Directions

We acknowledge that psychological adjustment has likely fluctuated along with the ebb and flow of COVID-19 cases and deaths in the United States broadly, and more specifically within states or counties that were hit particularly hard during the pandemic. Future research should examine whether the findings of this study can be replicated when accounting for county-level COVID rates and/or COVID-related restrictions (e.g., mandated social distancing or mask wearing). During the early stages of the pandemic, there was also misinformation perpetuated in the media that the pandemic was not something to be concerned about and that “normal” life would resume sooner rather than later. Thus, had data been collected later in the pandemic, it is possible we may have seen even less stability in subgroup membership over time. Future studies should extend our findings by examining changes in patterns of psychological adjustment from pre-pandemic to later on in the pandemic. Additionally, recent research indicates the important role of prior experiences of adversity in influencing psychological responses to pandemic-related stress [67]. Thus, it will be important for future research to determine the degree to which factors such as adverse experiences may contribute to these individuals’ maladjustment in order to identify recommendations that will enhance their well-being. We underscore that our data reflect a sample with minimal representation from many socially disadvantaged groups who have been disproportionately impacted by the pandemic; thus, more representative studies are needed to fully understand how and for whom HAI is beneficial or disadvantageous in the context of this public health crisis. This is particularly important given increasing evidence that the benefits and risk of HAI may be most pronounced among populations that experience adversity [68,69]. Although the LAPS is culturally appropriate for assessing attachment to pets among participants in our sample, future studies should consider its relevance for estimating attachment in culturally diverse populations.

Specific to HAI research, it may also be worthwhile to examine profiles of adjustment that include indicators of psychological resilience and well-being, given that the benefits of HAI include promoting self-esteem, self-efficacy, and other aspects of psychological resilience. Moreover, it is recommended that future studies examine other domains of HAI, such as comfort derived from pets, caretaking behaviors, pet-related stress, and physical touch, as moderators of patterns of adjustment. It may also be important to consider how change in attachment to pets is associated with changes in mental health during this public health crisis. Most likely, this is a bidirectional and reciprocal relationship that occurs over time, which cannot be examined with retrospective data. This is important given recent findings regarding the role of other domains of HAI (i.e., comfort from pets, positive engagement with pets) in buffering the negative impacts of various forms of stress on adjustment and in promoting personal hardiness and self-esteem [25,68,69,70]. We recommend that future studies also consider how individuals’ access to pet-related resources (e.g., pet pantry service, low-cost veterinary services [71]) may have influenced changes in psychological stress during this time, and believe this is an important direction for future research. In addition, it is important to explore whether HAI moderates transitions in latent patterns of adjustment among children and adolescents.

## 5. Conclusions

This study is unique in that it used a person-centered approach to investigate change in patterns of adults’ psychological adjustment during the initial months of the COVID-19 pandemic, and how changes in adjustment were influenced by attachment to pets. Our results indicate that stronger attachment to pets may have functioned as a protective factor for people with moderate and high levels of mental health symptoms prior to the COVID-19 pandemic; however, those with severe symptom profiles and high attachment to pets fared worst in the context of COVID-19 restrictions. Our study highlights the importance of utilizing person-centered approaches when investigating psychological adjustment over time. Studies investigating the impact of human–animal interactions on mental health should consider how attachment to pets may impact patterns of psychological adjustment, rather than using a variable-centered approach. Further, this study has important implications for those providing support services to pet owners during this COVID-19 pandemic. In particular, our results suggest that while interactions with pets may be beneficial for most pet owners (e.g., those with a moderate symptom profile), we should take caution when discussing pets as a solution to mental health concerns resulting from social isolation for those with severe psychological maladjustment. Mental health services should consider the impact of pet-related stressors on mental health during the COVID-19 pandemic and target those that may be most impacted by these additional stressors. In particular, low-resourced pet owners may be experiencing comparatively more severe hardships related to pet caregiving than their high-resourced counterparts. For example, the COVID-19 pandemic has exacerbated inequalities in housing in the U.S. and securing pet-friendly rental housing was already difficult for low-income individuals [72]. Our findings suggest that providing community-level support and public policy to help keep individuals and families together with their animal companions through hardship is in the interest of public mental health.

## Figures and Tables

**Table 1:** Demographic Characteristics of Sample (*n* = 1942).

Variable Name	Variable Categories	Frequency (%)
Race/Ethnicity	Arab/Arab American	2 (0.1)
	Asian/Asian American	42 (2.2)
	Black/African American	15 (0.8)
	First Nations/Indigenous	3 (0.2)
	Latino/Latina/Latinx	50 (2.6)
	South Asian/Pacific Islander	7 (0.4)
	White	1702 (87.6)
	Multiracial/Mixed Race	111 (5.7)
	Prefer to self-describe	10 (0.5)
Gender Identity	Cisgender female/woman	1743 (89.8)
	Cisgender male/man	137 (7.0)
	Genderqueer/gender non-conforming	21 (1.1)
	Transgender female/woman	2 (0.1)
	Transgender male/man	9 (0.5)
	Multiple Identities	27 (1.4)
	Missing	5 (0.3)
Sexual Orientation	Asexual	25 (1.3)
	Bisexual	157 (8.1)
	Demisexual	11 (0.6)
	Gay	21 (1.1)
	Heterosexual/straight	1510 (77.8)
	Lesbian	50 (2.6)
	Pansexual	27 (1.4)
	Queer	29 (1.5)
	Two-Spirit	1 (0.1)
	Prefer to self-describe	6 (0.3)
	Multiple identities	83 (4.3)
	Not sure/questioning	22 (1.1)
Employment Change	Begun new job	13 (0.7)
	Laid off or fired	121 (6.2)
	No change	680 (35.0)
	Working from home	692 (35.6)
	Other change	194 (10.0)
	Multiple options selected	242 (12.5)
Pet Type—Owned ^1^	Bird(s)	70 (3.6)
	Cat(s)	1029 (53.0)
	Dog(s)	1442 (74.3)
	Fish	155 (8)
	Horse(s)	54 (2.8)
	Small mammal(s) (e.g., rat, hedgehog, rabbit)	68 (3.5)
	Reptile(s) (e.g., snake, lizard, turtle)	109 (5.6)
	Other pet(s) (e.g., cow, goat, spider, chicken)	49 (2.5)
Pet Type—Favorite	Cat	667 (34.3)
	Dog	1275 (65.7)

^1^ Participants were able to report on all the pets that they lived with. These categories are not mutually exclusive.

**Table 2:** Intercorrelations, Unstandardized Means, and Standard Deviations of Continuous Key Variables.

Variable	1	2	3	4	5	6	7	8	9	10	11	12	13	14	15	16	17	18
1. Age	--																	
2. Attachment to Pet	−0.09	--																
3. Anxiety (T1)	−0.26	0.09	--															
4. Depression (T1)	−0.24	0.09	0.62	--														
5. Hostility (T1)	−0.22	0.09	0.51	0.52	--													
6. Interpersonal Sensitivity (T1)	−0.30	0.09	0.61	0.68	0.52	--												
7. Obsessive-Compulsive (T1)	−0.26	0.08	0.62	0.64	0.52	0.62	--											
8. Phobic Anxiety (T1)	−0.21	0.12	0.50	0.48	0.37	0.50	0.47	--										
9. Somatization (T1)	−0.08	0.08	0.46	0.43	0.37	0.40	0.47	0.35	--									
10. Additional Items (T1)	−0.10	0.07	0.50	0.53	0.36	0.47	0.54	0.38	0.43	--								
11. Anxiety (T2)	−0.24	0.09	0.62	0.46	0.34	0.45	0.43	0.39	0.34	0.37	--							
12. Depression (T2)	−0.30	0.09	0.50	0.68	0.38	0.54	0.47	0.38	0.34	0.41	0.66	--						
13. Hostility (T2)	−0.27	0.06	0.41	0.41	0.56	0.40	0.38	0.29	0.26	0.29	0.52	0.53	--					
14. Interpersonal Sensitivity (T2)	−0.28	0.13	0.47	0.55	0.40	0.73	0.49	0.42	0.34	0.38	0.52	0.63	0.49	--				
15. Obsessive-Compulsive (T2)	−0.31	0.07	0.53	0.53	0.40	0.50	0.69	0.39	0.36	0.45	0.60	0.65	0.51	0.55	--			
16. Phobic Anxiety (T2)	−0.12	0.07	0.34	0.28	0.23	0.31	0.28	0.42	0.20	0.26	0.55	0.46	0.30	0.36	0.41	--		
17. Somatization (T2)	−0.08	0.08	0.38	0.37	0.29	0.34	0.37	0.30	0.68	0.33	0.48	0.46	0.34	0.39	0.44	0.31	--	
18. Additional Items (T2)	−0.18	0.06	0.44	0.45	0.30	0.41	0.44	0.34	0.33	0.64	0.57	0.60	0.43	0.46	0.57	0.41	0.42	--
Mean	39.68	79.93	1.24	1.29	1.26	1.32	1.42	0.89	0.96	1.38	1.46	1.58	1.30	1.11	1.64	1.73	1.01	1.67
Standard Deviation	13.61	9.08	0.77	0.87	0.76	0.92	0.77	0.92	0.80	0.87	0.88	0.95	0.88	0.99	0.90	1.21	0.88	0.91

Note: All correlations between the continuous key variables are significant (*p* < 0.05).

**Table 3:** Fit Indices for Unconstrained Pre-COVID LPA Models for Each Variance–Covariance Structure.

Variance/Covariance Structure	*k*	Par	LL	AIC	BIC	aBIC	VLMR-LRT	LMR-LRT	BLRT	Entropy	Smallest Class		Condition #
							*p-*Value	*p-*Value	*p-*Value		*n*	%	
Non-Diagonal, Class Invariant	1	44	−16,322.3	32,732.5	32,979.4	32,839.7	NA	NA	NA	NA	2021	100%	2.55 × 10^−3^
	2	53	−16,194.0	32,494.0	32,791.4	32,623.0	0.429	0.432	0.000	0.841	241	12%	4.10 × 10^−4^
	3	62	−15,886.7	31,897.4	32,245.3	32,048.3	0.006	0.007	0.000	0.970	269	13%	1.74 × 10^−10^
	4	71	−15,177.5	30,496.9	30,895.3	30,669.8	0.000	0.000	0.000	0.984	115	6%	2.73 × 10^−10^
Diagonal, Class Invariant	1	16	−20,027.8	40,087.5	40,177.3	40,126.5	NA	NA	NA	NA	2021	100%	3.63 × 10^−2^
	2	25	−17,758.9	35,567.8	35,708.0	35,628.6	0.000	0.000	0.000	0.888	482	24%	1.92 × 10^−2^
	3	34	−16,789.1	33,646.2	33,837.0	33,728.9	0.000	0.000	0.000	0.855	286	14%	5.04 × 10^−3^
	4	43	−16,438.3	32,962.7	33,204.0	33,067.3	0.021	0.022	0.000	0.846	150	7%	1.33 × 10^−3^
	**5**	**52**	**−16,277.8**	**32,659.5**	**32,951.3**	**32,786.1**	**0.000**	**0.000**	**0.000**	**0.809**	**91**	**5%**	**5.41 × 10^−4^**
	6	61	−16,221.7	325,65.3	32,907.6	32,713.8	0.318	0.325	0.000	0.826	95	5%	2.77 × 10^−6^
Diagonal, Class Varying	1	16	−20,027.8	40,087.5	40,177.3	40,126.5	NA	NA	NA	NA	2021	100%	3.63 × 10^−2^
	2	33	−17,582.1	35,230.3	35,415.5	35,310.6	0.000	0.000	0.000	0.848	640	32%	1.21 × 10^−2^
	3	50	−16,349.1	32,798.2	33,078.8	32,920.0	0.000	0.000	0.000	0.860	528	26%	3.14 × 10^−3^
Non-Diagonal, Class Varying	1	44	−16,322.3	32,732.5	32,979.4	32,839.7	NA	NA	NA	NA	2021	100%	2.55 × 10^−3^
	2	89	−15,748.0	31,674.0	32,173.4	31,890.6	0.000	0.000	0.000	0.567	872	43%	3.82 × 10^−4^
	3	134	−15,646.4	31,560.9	32,312.8	31,887.1	0.107	0.108	0.000	0.700	111	5%	8.12 × 10^−5^

Note: *n* = 2021; Bolded row indicates the model that was selected for further analysis; *k* = number of classes; Par = number of parameters; AIC = Akaike information criterion; BIC = Bayesian Information Criterion; aBIC = sample-adjusted BIC; VLMR-LRT = Vuong-Lo-Mendell-Rubin likelihood ratio test; LMR-LRT = Lo-Mendell-Rubin likelihood ratio test; BLRT = Bootstrap likelihood ratio test; LRTs and Entropy not applicable for 1-class model.

**Table 4:** Fit Indices for Unconstrained Post-COVID LPA Models for Each Variance–Covariance Structure.

Variance/Covariance Structure	*k*	Par	LL	AIC	BIC	aBIC	VLMR-LRT	LMR-LRT	BLRT	Entropy	Smallest Class		Condition #
							*p-*Value	*p-*Value	*p-*Value		*n*	%	
Non-Diagonal, Class Invariant	1	44	−18,177.9	36,443.8	36,690.2	36,550.4	NA	NA	NA	NA	2000	100%	1.88 × 10^−3^
	2	53	−18,064.4	36,234.8	36,531.6	36,363.3	0.000	0.000	0.000	0.779	317	16%	1.56 × 10^−3^
	3	62	−17,873.2	35,870.4	36,217.6	36,020.7	0.002	0.002	0.000	0.952	181	9%	4.88 × 10^−9^
	4	71	−17,449.5	35,040.9	35,438.6	35,213.0	0.000	0.000	0.000	0.971	169	8%	9.40 × 10^−12^
	5	80	−17,340.4	34,840.8	35,288.9	35,034.7	0.027	0.028	0.000	0.956	42	2%	6.72 × 10^−14^
	6	89	−17,101.6	34,381.3	34,879.8	34,597.0	0.210	0.214	0.000	0.977	12	1%	7.31 × 10^−16^
Diagonal, Class Invariant	1	16	−21,822.8	43,677.6	43,767.2	43,716.4	NA	NA	NA	NA	2000	100%	1.73 × 10^−2^
	2	25	−19,516.7	39,083.4	39,223.5	39,144.0	0.000	0.000	0.000	0.860	672	34%	3.46 × 10^−2^
	3	34	−18,651.1	37,370.3	37,560.7	37,452.7	0.000	0.000	0.000	0.844	267	13%	9.08 × 10^−3^
	4	43	−18,309.1	36,704.3	36,945.1	36,808.5	0.000	0.000	0.000	0.833	198	10%	2.62 × 10^−3^
	**5**	**52**	**−18,203.7**	**36,511.5**	**36,802.7**	**36,637.5**	**0.036**	**0.037**	**0.000**	**0.823**	**80**	**4%**	**6.78 × 10^−4^**
	6	61	−18,122.4	36,366.9	36,708.5	36,514.7	0.002	0.002	0.000	0.796	77	4%	5.03 × 10^−4^
	7	70	−18,034.2	36,208.5	36,600.5	36,378.1	0.412	0.418	0.000	0.861	92	4.6%	2.77 × 10^−6^
Diagonal, Class Varying	1	16	−21,822.8	43,677.6	43,767.2	43,716.4	NA	NA	NA	NA	2000	100%	1.73 × 10^−2^
	2	33	−19,350.8	38,767.6	38,952.4	38,847.6	0.000	0.000	0.000	0.846	820	41%	2.14 × 10^−2^
	3	50	−18,510.2	37,120.4	37,400.5	37,241.6	0.020	0.021	0.000	0.841	310	16%	8.48 × 10^−3^
Non-Diagonal, Class Varying	1	44	−18,177.9	36,443.8	36,690.2	36,550.4	NA	NA	NA	NA	2000	100%	1.88 × 10^−3^

Note: *n* = 2021; Bolded row indicates the model that was selected for further analysis; *k* = number of classes; Par = number of parameters; AIC = Akaike information criterion; BIC = Bayesian Information Criterion; aBIC = sample-adjusted BIC; VLMR-LRT = Vuong-Lo-Mendell-Rubin likelihood ratio test; LMR-LRT = Lo-Mendell-Rubin likelihood ratio test; BLRT = Bootstrap likelihood ratio test; LRTs and Entropy not applicable for 1-class model.

**Table 5:** Transition Probabilities and Logistic Regression Coefficients for the Effects of Predictors on Transition Probabilities.

		Post-COVID				
		Low Symptoms (12%)	Mild Symptoms (42%)	Moderate Symptoms (32%)	High Symptoms (11%)	Severe Symptoms (4%)
Pre-COVID	Transition probabilities					
	Low symptoms (12%)	0.67	0.23	0.08	0.03	0.00
	Mild symptoms (39%)	0.02	0.81	0.14	0.03	0.00
	Moderate symptoms (33%)	0.00	0.09	0.79	0.12	0.01
	High symptoms (11%)	0.00	0.04	0.23	0.70	0.03
	Severe symptoms (5%)	0.01	0.06	0.20	0.34	0.39
	Transition proportions (%)					
	Low symptoms (12%)	10.14%	1.34%	0.36%	0.05%	0.00%
	Mild symptoms (39%)	1.85%	33.52%	3.50%	0.77%	0.26%
	Moderate symptoms (33%)	0.26%	3.50%	25.49%	3.30%	0.36%
	High symptoms (11%)	0.00%	0.46%	2.21%	7.11%	0.82%
	Severe symptoms (5%)	0.05%	0.21%	0.57%	0.88%	2.99%
	Attachment to pets (X^2^)					
	Low symptoms (12%)	^a^	−0.87	0.35	16.06 ^b^	0.26 ^b^
	Mild symptoms (39%)	^a^	−0.21	−0.23	18.52	0.79
	Moderate symptoms (33%)	^a^	−12.51 ***	−13.18 ***	−13.35 ***	−12.02 ***
	High symptoms (11%)	^a^	−5.34 ^b^	−4.13 ***	−4.79 ***	−5.76 ***
	Severe symptoms (5%)	^a^	−22.29 ^b^	−22.83 ***	−22.58 ***	−21.37 ***

Note: *** *p* < 0.001; ^a^ reference group; ^b^ the significance of the effect could not be determined, as some of the multinomial logit parameters were fixed within the model to avoid singularity of the information matrix; logistic regression analyses controlled for age, race/ethnicity, LGBTQIA+ identities, relationship status, and employment status.

## Data Availability

The data presented in this study are available on request from the 1st Author (Shelby E. McDonald). The data are not publicly available due to participant confidentiality.
